# Methodological advances in claims-based dementia algorithms: integrating medication and clinical data for medicare populations

**DOI:** 10.1186/s12874-026-02929-7

**Published:** 2026-06-25

**Authors:** Sara Knox, Janet Horn, Kit Simpson, Bethany Wolf

**Affiliations:** 1 Department of Health Sciences and Research, College of Health Professions, Medical University of South Carolina, 77 President St, MSC 700, Charleston, SC 29425 USA; 2 Ralph H Johnson Veterans Affairs Health Care System, 109 Bee St, Charleston, SC 29425 USA; 3 Department of Healthcare Leadership & Management, College of Health Professions, Medical University of South Carolina, 151 Rutledge Ave, Charleston, SC 29425 USA; 4 Department of Public Health Sciences, College of Medicine, Medical University of South Carolina, 135 Cannon St, Charleston, SC 29425 USA

**Keywords:** Alzheimer’s disease and related dementias, Medicare, Digital algorithm, Predictive modeling

## Abstract

**Background:**

Accurate identification of Alzheimer’s Disease and Related Dementias (ADRD) in Medicare data is essential for research, policy, and care planning. Existing approaches, including the Centers for Medicare & Medicaid Services (CMS) Chronic Conditions Warehouse (CCW) algorithm, rely primarily on International Classification of Diseases, Tenth Revision (ICD-10) codes and are subject to misclassification and potential bias across populations.

**Methods:**

We evaluated whether incorporating neuropsychiatric symptom (NPS) ICD-10 codes and medication data improves prediction of algorithm-defined ADRD classificaitons. We analyzed 269,214 Medicare beneficiaries receiving home health services in 2019 using claims, assessment, and prescription drug files. Two classification algorithms were examined: (1) the CCW binary classification algorithm and (2) a clinically informed ADRD ternary classification algorithm (ADRD-highly likely, ADRD-possible, ADRD-unlikely). Predictive models were developed for each algorithm using stacked elastic net regression, incorporating demographics, healthcare utilization, psychiatric and cognitive indicators, and medication use. Models were trained on beneficiaries with definitive ADRD status and evaluated in an independent test set using area under the curve (AUC), sensitivity, specificity, and predictive values. Thresholds were selected to prioritize specificity (≥ 80%) while maximizing sensitivity.

**Results:**

In the test dataset, the CCW predictive model achieved an AUC of 0.863 for predicting CCW binary-defined ADRD status. The ADRD predictive model achieved a higher AUC of 0.906 when predicting ADRD ternary-defined classifications. Both models demonstrated strong discrimination for their respective outcomes, but performance characteristics differed. Among beneficiaries classified as ADRD-possible, the ADRD predictive model more frequently classified individuals as ADRD-negative, indicating a more conservative approach to ambiguous cases.

**Conclusions:**

Incorporating NPS codes and medication data improves prediction of algorithm-defined ADRD classifications, particularly among individuals with uncertain status. Models aligned with the ADRD ternary algorithm demonstrated more specific and conservative classification patterns, suggesting that integrating behavioral, cognitive, and medication data may enhance the validity of claims-based phenotyping. Further validations against clinical diagnosis are needed.

**Supplementary Information:**

The online version contains supplementary material available at https://doi.org/10.1186/s12874-026-02929-7.

## Background

Currently, nearly 7 million Americans are living with Alzheimer’s Disease or Related Dementias (ADRD), a number projected to exceed 12 million by 2050, with annual costs approaching $1 trillion by mid-century [1]. The burden of ADRD for patients, families, and society underscores the urgent need for accurate identification methods to guide care, research, and policy. Medicare data provide a unique opportunity to examine real-world evidence for older adults, including people with ADRD. However, reliance on International Classification of Diseases (ICD-10) codes in the Centers for Medicare and Medicaid Services (CMS) Chronic Conditions Warehouse (CCW) binary classification algorithm for ADRD has well-documented limitations. Studies comparing claims-based definitions with clinically adjudicated diagnoses have identified both false positives and false negatives, as well as disparities in ADRD classification across racial and ethnic groups [2–8]. Beyond compromising research validity, such misclassification can have serious consequences for patients and families. False negatives may delay recognition of ADRD in research datasets, while false positives may introduce bias in studies relying on administrative classifications.

The CCW binary classification algorithm assigns and ADRD-positive status when a single claim includes one of 22 ICD-10 codes within a three-year look back period. Beneficiaries who do not meet these criteria are classified as ADRD-negative. This rule-based approach prioritizes sensitivity but may capture provisional or billing-driven diagnoses rather than confirmed clinical dementia, limiting validity for research applications [4, 6]. Prior strategies used to refine claims-based algorithms include restricting ICD-10 codes used to define ADRD, requiring multiple claims with evidence of an ADRD diagnosis, or incorporating comorbidities. However, these strategies remain constrained by coding limitations, relying solely on billing data that often reflect reimbursement practices more than actual clinical diagnoses of dementia.

To address the limitations of the CCW binary classification algorithm, a recently developed ADRD ternary classification algorithm developed by our team, applies a three-tiered classification for dementia likelihood [9]. It incorporates a shortened, clinically adjudicated list of ICD-10 codes, requires multiple episodes within a 1-year period, and applies exclusionary criteria based on co-occurring conditions that complicate dementia diagnosis. This approach classifies individuals as ADRD-highly likely, ADRD-possible, or ADRD-unlikely, aiming to improve accuracy over binary algorithms.

Although, the ADRD ternary classification algorithm refines diagnostic code criteria and applies exclusionary conditions, it shares a key limitation with the CCW binary classification algorithm: both rely primarily on ADRD diagnosis codes. Neither incorporates information on medications or neuropsychiatric symptoms, which could substantially improve dementia identification. Neuropsychiatric symptoms (NPS) occur in up to 98% of ADRD patients [10] and often prompt pharmacologic treatment, so prescription records in Medicare Part D can complement diagnosis codes to enhance phenotyping. Home health (HH) data, used by approximately one-third of community-dwelling persons with ADRD [1, 11–14] offer detailed cognitive, psychiatric, and functional information rarely available in claims. Administrative data are subject to detection bias stemming from disparities in healthcare access and utilization, particularly among marginalized populations [15]. Incorporating medications, NPS information, ad HH data may improve ascertainment and partially mitigate these biases. Enhanced identification methods may help support more equitable representation in research, while acknowledging the limitations of the underlying data sources. Therefore, this study evaluates whether NPS codes and medication data, key signals not incorporated into existing algorithms, can improve the prediction of algorithm-defined ADRD classifications, particularly among individuals whose ADRD status is ambiguous.

## Methods

### Study design and population

**Fig. 1:**
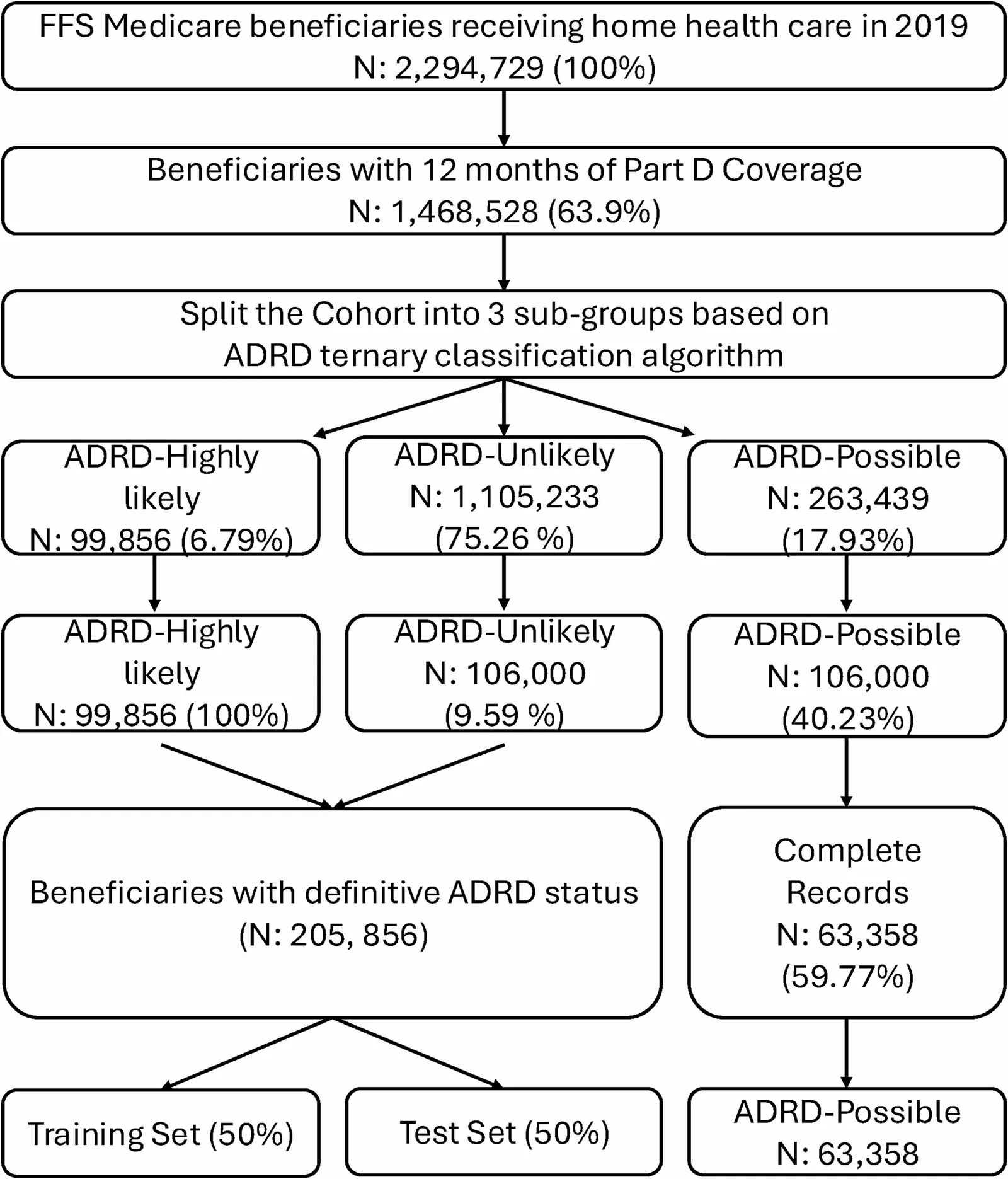
Cohort selection and construction of analytic samples. (Legend) Beneficiaries receiving home health services in 2019 with continuous Medicare Part D enrollment were identified and classified using the ADRD ternary classification algorithm. A stratified random sample was used to create the analytical cohort. Beneficiaries with definitive ADRD status (ADRD-highly likely or ADRD-unlikely) were split into training and test datasets, while those classified as ADRD-possible were retained for separate analyses after removing individuals who had missing variables. (Abbreviations: ADRD = Alzheimer’s disease and related dementias; CCW = Chronic Conditions Warehouse; FFS = Fee-for-Service)

We conducted a retrospective, observational cohort study using 2019 Medicare data. The target population included fee-for-service (FFS) beneficiaries aged ≥ 64 who received HH services. Beneficiaries receiving HH services in 2019 were identified and restricted to those with 12 months of continuous Medicare Part D enrollment to ensure availability of prescription data. Individuals were initially classified using the ADRD ternary classification algorithm (ADRD-highly likely, ADRD-possible, ADRD-unlikely). To achieve approximately balanced representation across ADRD classification strata, we randomly sampled ~approximately 100,000 individuals from the ADRD-possible and ADRD-unlikely groups, while retaining all ADRD-highly likely beneficiaries due to smaller sample size. A total of 311,856 beneficiaries were initially extracted. Beneficiaries were grouped according to ADRD status, with those classified as ADRD-highly likely or ADRD-unlikely considered “definitive” and combined for the main analytical cohort (205, 856). The definitive-status group was then randomly split into a 50% training and a 50% test datasets. The ADRD-possible group was retained separately for sub-analyses. Individuals in the ADRD-possible group who were missing study variables were removed prior to sub-analyses (63,358). Cohort selection and the construction of the analytical dataset is presented in Fig. 1.

### Data sources

Five CMS files were used: (1) Outcomes and Assessment Information Set (OASIS; clinical assessment data) (2), Home Health Agency Fee-for-Service (FFS) files (claims data) (3), Medicare Provider Analysis and Review (MedPAR; utilization data) (4), Medicare Beneficiary Summary Files (MBSF; enrollment data), and (5) Part D event files (prescription fill data).

### Outcomes

To clarify terminology, we use the term “classification algorithm” to refer to the rule-based systems used to assign ADRD status from administrative and clinical data, specifically the CCW binary classification algorithm and the ADRD ternary classification algorithm. These algorithm-derived classifications served as the outcome variables for predictive models.

The CCW algorithm (version 27) is a binary classification (ADRD-positive/ADRD-negative). It classifies beneficiaries as ADRD-positive if, within a 3-year look-back period, they have at least one inpatient, skilled nursing, HH, hospital outpatient, or carrier claim with one of twenty-two ICD-10 diagnosis codes for dementia. Beneficiaries who do not meet these criteria are classified as ADRD-negative. This algorithm classifies beneficiaries on a ternary scale: ADRD-highly likely, ADRD-possible, or ADRD-unlikely. Beneficiaries are classified as ADRD-highly likely if, within a 1-year lookback, they have ≥ 2 records in MedPAR or HH files with one of 12 validated ICD-10 codes for dementia and no exclusionary conditions. The ICD-10 code list was derived from clinician-adjudicated research [5], and exclusionary criteria reflect comorbidities that preclude a definitive ADRD diagnosis (e.g., preceding stroke, or concurrent psychiatric diagnosis) [16, 17]. Beneficiaries meeting partial criteria (e.g., one qualifying ICD-10 code, or two codes plus exclusionary comorbidity) are classified as ADRD-possible. Those without any qualifying ICD-10 dementia codes are classified as ADRD-unlikely. A complete list of ICD-10 codes, exclusionary conditions, and classification logic for the ADRD ternary classification algorithm is provided in Supplementary Tables 1 and a schematic representation of the ADRD ternary classification algorithm is shown in Supplemental Fig. 1.

### Variables

Covariates included demographic characteristics (age, sex, race/ethnicity, marital status, education, Medicare entitlement reason, dual eligibility), healthcare utilization (acute care, skilled nursing, emergency department visits, hospice stays, readmissions), psychiatric and cognitive symptoms (confusion, anxiety, depression, functional dependence, agitation, executive dysfunction, apraxia), and prescription use [any fill or continuous use (≥ 2 fills) for ADRD or psychiatric medications; Table 1). Comorbidities were measured using Elixhauser categories [18]. 

**Table 1:** Medications Used for Alzheimer’s Disease and Neuropsychiatric Symptoms

Condition	Medication (Brand name)	Classification
Alzheimer’s Disease and Related Dementias		
Cognition	Aducanumab (Aduhelm)	Amyloid beta-directed monoclonal antibody
	Donepezil hydrochloride (Aricept)	Cholinesterase inhibitor
	Rivastigmine (Exelon)	Acetylcholinesterase Inhibitors
	Galantamine hydrobromide (Razadyne)	Acetylcholinesterase Inhibitors
	Memantine (Namenda)	NMDA receptor antagonists.
	Donepezil and Memantine (Namzaric)	NMDA receptor antagonist, acetylcholinesterase inhibitor
Neuropsychiatric Symptom Medications		
Anxiety	Lorazepam (Ativan)	Benzodiazepines
	Buspirone (BuSpar)	Antianxiety Agents, Anxiolytics, Nonbenzodiazepines.
	Prazepam (Centrax)	Benzodiazepine
	Propanolol (Inderal)	Antidysrhythmics, II, Beta-Blockers, Antianginal
	Clonazepam (Klonopin)	Benzodiazepine
	Escitalopram (Lexapro)	Selective serotonin reuptake inhibitors (SSRI)
	Chlordiazepoxide (Librium)	Anxiolytics, Benzodiazepines
	Oxazepam (Serax)	Benzodiazepine
	Atenolol (Tenormin)	Beta-Blockers, Beta-1 Selective
	Clorazepate (Tranxene)	Benzodiazepine
	Diazepam (Valium)	Benzodiazepine
	Alprazolam (Xanaz)	Benzodiazepine
Insomnia	Suvorexant (Belsomra)	Sedatives or hypnotics
Depression	Fluoxetine (Prozac)	Selective serotonin reuptake inhibitors
	Citalopram (Celexa)	Selective serotonin reuptake inhibitors
	Sertraline (Zoloft)	Selective serotonin reuptake inhibitors
	Paroxetine (Paxil)	Selective serotonin reuptake inhibitors
	Escitalopram (Lexapro)	Selective serotonin reuptake inhibitors
	Venlafaxine (Effexor)	Serotonin-norepinephrine reuptake inhibitor
	Duloxetine (Cymbalta)	Selective serotonin and norepinephrine reuptake inhibitors
	Bupropion (Wellbutrin)	Antidepressants, Dopamine Reuptake Inhibitors

Legend: Medication classes commonly used in the management of Alzheimer’s disease and related dementias (ADRD) and associated neuropsychiatric symptoms, including cognitive enhancers and agents used to treat anxiety, insomnia, and depression. Brand names are provided in parentheses. Classification reflects the primary pharmacologic mechanism or therapeutic category

### Statistical analysis

We conducted a multistep analytic process designed to optimize variable selection, reduce overfitting, and rigorously evaluate model performance [19, 20]. The analytic dataset for model development included 205,856 beneficiaries with definitive ADRD status (ADRD-highly likely or ADRD-unlikely, excluding ADRD-possible individuals). This analytical dataset was randomly split into training (50%) and test (50%) datasets. The training dataset was used to develop predictive models, while the test dataset was reserved for independent evaluation of model performance. Beneficiaries with ADRD-possible status were excluded from the analytic dataset and model training because their classification is inherently uncertain. Sub-analyses were run separately on this sub-group. This ensures that model development was based on definitive ADRD cases while allowing evaluation of model performance in ambiguous cases.

Continuous variables (e.g., age, healthcare utilization) were standardized before model fitting. Categorical variables were dummy-coded. Missing data were common for cognitive and psychiatric measures (16–35%) and moderate for readmissions and functional dependence (20–25%). Missing data were addressed using multiple imputation by chained equations (MICE) with 25 imputations in the training dataset. The test dataset was imputed separately using a single chained equations approach.

Predictive models were developed to estimate the probability of algorithm-defined ADRD status based on selected predictors. In this framework, the classification algorithms determines the rule-based ADRD labels, and the predictive model estimates the probability that a beneficiary would receive a given algorithm-defined classification.

Two models were estimated: (1) a CCW predictive model, with outcome defined by the CCW binary classification algorithm (ADRD-positive vs. ADRD-negative), and (2) an ADRD predictive model, with the outcome defined by the ADRD ternary classification algorithm (ADRD-highly likely, ADRD-possible, or ADRD-unlikely).

Candidate predictors included demographic variables, prior healthcare utilization, psychiatric and cognitive symptoms, and medication use (any vs. repeated prescriptions). Variable selection was performed using stacked adaptive elastic net regression (saenet in R *miselect* package), which enables simultaneous selection across multiply imputed datasets while reducing bias from collinearity [19]. Because each model was trained on a different algorithm-defined outcome, the selected predictor sets were allowed to differ across models. Shared predictors across the models reflect consistent associations with ADRD status, while model-specific predictors reflect algorithm-specific signals.

To asses the contribution of key predictor domains, we conducted ablation analyses by refitting models excluding [1] medication variables [2], OASIS-based functional and cognitive indicators, and [3] both sets of variables (collectively referred to as reducted models).

Penalty terms (α and λ) were tuned using 10-fold cross-validation within the training dataset, with optimal values selected using the 1-SE rule to favor more parsimonious models. Final predictor sets were then entered into multivariable logistic regression models estimated across each imputed dataset. Full model coefficient and reduced model coefficients are provided in Supplemental Tables 2 and 3.

Each final model was applied to an independent test dataset consisting of 50% of the analytic cohort, which was not used during model development. Model fit in the training data was evaluated using pooled Akaike Informaiton Criteria (AIC) and pooled adjusted pseudo R^2^ across imputations [21]. 

Predictive performance in the test dataset was primarily assessed using the area under the receiver operating characteristic curve (AUC). Additional metrics included sensitivity, specificity, positive predictive value (PPV), and negative predictive value (NPV). To prioritize specificity, thresholds were selected to maintain specificity ≥ 80%, with the optimal threshold defined as the point that maximized sensitivity under this constraint.

Agreement between models was evaluated as the proportion of concordant predictions. AUCs were also compared between beneficiaries with concordant versus discordant predictions. Model performance was further evaluated using calibration metrics (calibration slope and intercept) and visual calibration plots. Given the large sample size, interpretation focused on effect size magnitude and direction rather than statistical significance.

Both models were applied to the subgroup of 63,358 beneficiaries with ADRD-possible status under the ADRD ternary classification algorithm. We examined the post-hoc proportion of beneficiaries with definitive by the CCW predictive model (ADRD-positive or ADRD-negative), and by the ADRD predictive model (ADRD-highly likely and ADRD-unlikely), as well as concordance between model predictions (CCW binary classification algorithm ADRD-positive & ADRD ternary classification algorithm ADRD-highly likely; CCW binary classification algorithm ADRD-negative & ADRD ternary classification algorithm ADRD-unlikely). Predicted probability distributions were compared graphically. All analyses were conducted in R v. 4.3.2 [22]. 

## Results

### Cohort characteristics

There were 269,214 beneficiaries included in the analytical cohort. Among the analytic cohort, 205,856 beneficiaries had a definitive ADRD status (99,856 ADRD-positive and106,000 ADRD-negative) and 63,358 beneficiaries with an ADRD-possible classification. There was strong agreement between the ADRD ternary classification algorithm and the CCW binary classification algorithm, with 82.6% (170,084) having concordant status (87,458 ADRD-positive/ADRD-highly likely; 82,828 ADRD-negative/ADRD-unlikely). Participant characteristics by ADRD classifications for each algorithm are shown in Table 2 (CCW binary classification algorithm) and Table 3 (ADRD ternary classification algorithm).

**Table 2:** Baseline Characteristics by Chronic Condition Warehouse Binary Classification

	ADRD-Negative (*N* = 127460)	ADRD-Positive (*N* = 143734)
Age, Years, mean (SD)	78.752 (8.362)	84.654 (7.612)
Sex, Female, n (%)	82,865 (65.013)	96,382 (67.056)
Race, n (%)		
White	95,805 (75.165)	112,348 (78.164)
Black	13,642 (10.703)	14,474 (10.07)
Other	18,013 (14.132)	16,912 (11.766)
Dual eligibility, Yes, n (%)	43,703 (34.288)	44,074 (30.664)
Hospital outpatient visits, median (IQR)	8 (3, 19)	6 (2, 15)
Home health visits, median (IQR)	19 (10, 36)	27 (14, 49)
ER visits, median (IQR)	1 (0, 1)	1 (0, 2)
Acute inpatient hospital stays, median (IQR)	1 (0, 2)	1 (0, 2)
Skilled nursing facility stays, median (IQR)	0 (0, 1)	0 (0, 1)
Hospice stays, median (IQR)	0 (0, 0)	0 (0, 0)
Readmissions, median (IQR)	0 (0, 0)	0 (0, 0)
Prior functional cognition, Impaired, n (%)	30,377 (32.482)	67,621 (72.398)
Current Cognitive functioning, Impaired, n (%)	49,483 (42.937)	96,313 (78.35)
Confused, Yes, n (%)	56,739 (49.233)	101,318 (82.421)
Anxious, Yes, n (%)	52,049 (45.163)	66,409 (54.023)
Functional dependence, Yes, n (%)	49,137 (44.586)	79,646 (68.617)
Anxiety, Yes, n (%)	5733 (4.498)	4925 (3.426)
Wandering, Yes, n (%)	138 (0.108)	890 (0.619)
Impulse disorder, Yes, n (%)	114 (0.089)	205 (0.143)
Sleep disorder, Yes, n (%)	703 (0.552)	860 (0.598)
Amnesia, Yes, n (%)	2156 (1.692)	4212 (2.93)
Executive dysfunction, Yes, n (%)	74 (0.058)	102 (0.071)
Restlessness and agitation, Yes, n (%)	528 (0.414)	1624 (1.13)
Apathy, Yes, n (%)	6 (0.005)	8 (0.006)
Hostility, Yes, n (%)	2 (0.002)	7 (0.005)
Emotional liability, Yes, n (%)	34 (0.027)	54 (0.038)
Aphasia, Yes, n (%)	1995 (1.565)	3251 (2.262)
Agnosia, Yes, n (%)	5 (0.004)	16 (0.011)
Apraxia, Yes, n (%)	82 (0.064)	144 (0.1)
Depression, Yes, n (%)	44,442 (34.867)	66,345 (46.158)
# anxiety medications, median (IQR)	2 (0, 5)	1 (0, 4)
# depression medications, median (IQR)	0 (0, 1)	0 (0, 1)
# insomnia medications, median (IQR)	0 (0, 0)	0 (0, 0)
# Dementia medications, median (IQR)	0 (0, 0)	0 (0, 0)
Any anxiety medications, Yes, n (%)	18,763 (14.721)	22,059 (15.347)
Consistent anxiety medications, Yes, n (%)	12,984 (10.187)	15,600 (10.853)
Any depression medications, Yes, n (%)	7457 (5.85)	9719 (6.762)
Consistent depression medications, Yes, n (%)	5491 (4.308)	7652 (5.324)
Any insomnia medications, Yes, n (%)	21 (0.016)	42 (0.029)
Consistent insomnia medications, Yes, n (%)	8 (0.006)	20 (0.014)
Any dementia medications, Yes, n (%)	971 (0.762)	7262 (5.052)
Consistent dementia medications, Yes, n (%)	794 (0.623)	6152 (4.28)

Legend. Participant demographic, clinical, and utilization characteristics stratified by Alzheimer’s disease and related dementias (ADRD) status as determined by the CCW binary classification algorithm. Continuous variables are presented as mean (SD) or median (IQR), and categorical variables as n (%). ADRD-Negative and ADRD-Positive groups reflect CCW classification based on claims-derived indicators of cognitive impairment and dementia-related diagnoses. The analytic cohort includes 269,214 beneficiaries after application of inclusion criteria. The ADRD ‘possible’ group reflects individuals meeting criteria after exclusion rules specific to the ADRD ternary classification algorithm; this subgroup is not separately represented in the CCW classification

Abbreviations: Alzheimer’s Disease and Related Dementias *ADRD*

**Table 3:** Baseline Characteristics by Alzheimer’s Disease and Related Dementias Ternary Classification

	ADRD-Unlikely (*N* = 106000)	ADRD-highly Likely (*N* = 99856)	ADRD Possible (*N* = 65338)
Age, Years, mean (SD)	79.062 (8.342)	85.752 (7.160)	80.535 (8.428)
Sex, Female, n (%)	69,785 (65.835)	68,177 (68.275)	41,285 (63.187)
Race, n (%)			
White	82,902 (78.209)	72,307 (72.411)	52,944 (81.031)
Black	9841 (9.284)	12,100 (12.117)	6175 (9.451)
Other	13,257 (12.507)	15,449 (15.471)	6219 (9.518)
Dual eligibility, Yes, n (%)	32,939 (31.075)	35,759 (35.811)	19,079 (29.2)
Hospital outpatient visits, median (IQR)	8 (3, 19)	4 (1, 12)	9 (3, 22)
Home health visits, median (IQR)	18 (10, 35)	29 (15, 52)	22 (12, 39)
ER visits, median (IQR)	0 (0, 1)	1 (0, 1)	1 (1, 2)
Acute inpatient hospital stays, median (IQR)	1 (0, 2)	1 (0, 2)	2 (1, 3)
Skilled nursing facility stays, median (IQR)	0 (0, 1)	0 (0, 1)	1 (0, 1)
Hospice stays, median (IQR)	0 (0, 0)	0 (0, 0)	0 (0, 0)
Readmissions, median (IQR)	0 (0, 0)	0 (0, 0)	0 (0, 1)
Prior functional cognition, Impaired, n (%)	23,469 (31.438)	49,797 (83.969)	24,732 (46.695)
Current Cognitive functioning, Impaired, n (%)	37,637 (40.507)	69,979 (87.56)	38,180 (58.435)
Confused, Yes, n (%)	43,254 (46.553)	72,166 (90.297)	42,637 (65.256)
Anxious, Yes, n (%)	41,699 (44.879)	43,425 (54.335)	33,334 (51.018)
Functional dependence, Yes, n (%)	38,231 (43.811)	55,349 (75.123)	35,203 (53.878)
Anxiety, Yes, n (%)	5490 (5.179)	260 (0.26)	4908 (7.512)
Wandering, Yes, n (%)	149 (0.141)	783 (0.784)	96 (0.147)
Impulse disorder, Yes, n (%)	86 (0.081)	88 (0.088)	145 (0.222)
Sleep disorder, Yes, n (%)	488 (0.46)	438 (0.439)	637 (0.975)
Amnesia, Yes, n (%)	106 (0.1)	2371 (2.374)	3891 (5.955)
Executive dysfunction, Yes, n (%)	6 (0.006)	55 (0.055)	115 (0.176)
Restlessness and agitation, Yes, n (%)	374 (0.353)	1118 (1.12)	660 (1.01)
Apathy, Yes, n (%)	3 (0.003)	6 (0.006)	5 (0.008)
Hostility, Yes, n (%)	6 (0.006)	1 (0.001)	2 (0.003)
Emotional liability, Yes, n (%)	22 (0.021)	36 (0.036)	30 (0.046)
Aphasia, Yes, n (%)	1152 (1.087)	2078 (2.081)	2016 (3.085)
Agnosia, Yes, n (%)	4 (0.004)	10 (0.01)	7 (0.011)
Apraxia, Yes, n (%)	46 (0.043)	98 (0.098)	82 (0.126)
Depression, Yes, n (%)	39,915 (37.656)	37,646 (37.7)	33,226 (50.852)
# anxiety medications, median (IQR)	2 (1, 5)	1 (0, 3)	1 (0, 4)
# depression medications, median (IQR)	0 (0, 1)	0 (0, 1)	0 (0, 1)
# insomnia medications, median (IQR)	0 (0, 0)	0 (0, 0)	0 (0, 0)
# Dementia medications, median (IQR)	0 (0, 0)	0 (0, 1)	0 (0, 0)
Any anxiety medications, Yes, n (%)	17,101 (16.133)	12,392 (12.41)	11,329 (17.339)
Consistent anxiety medications, Yes, n (%)	12,213 (11.522)	8621 (8.633)	7750 (11.861)
Any depression medications, Yes, n (%)	6259 (5.905)	5936 (5.945)	4981 (7.623)
Consistent depression medications, Yes, n (%)	4627 (4.365)	4880 (4.887)	3636 (5.565)
Any insomnia medications, Yes, n (%)	23 (0.022)	26 (0.026)	14 (0.021)
Consistent insomnia medications, Yes, n (%)	9 (0.008)	14 (0.014)	5 (0.008)
Any dementia medications, Yes, n (%)	1020 (0.962)	6448 (6.457)	765 (1.171)
Consistent dementia medications, Yes, n (%)	815 (0.769)	5560 (5.568)	571 (0.874)

Legend. Demographic, clinical, and healthcare utilization characteristics stratified by classification under the ADRD ternary classification algorithm: ADRD-Highly likely, ADRD-Unlikely, and ADRD- possible. Continuous variables are presented as mean (SD) or median (IQR), and categorical variables as n (%). The ADRD ternary classification algorithm is based on diagnostic codes, frequency of claims, and exclusionary conditions; clinical and functional variables shown here are descriptive and not used in classification. The ADRD-possible classification group reflects individuals meeting criteria after application of ADRD specific exclusions

Abbreviations: Alzheimer’s Disease and Related Dementias *ADRD*

### Model description

The final logistic regression model selected for the CCW binary classification algorithm is presented in Table 4 and the final model for the ADRD ternary classification algorithm is shown in Table 5. Both predictive models included a core set of variables: age, sex, race (with minor differences in the reference categories used), dual eligibility status, number of hospital outpatient visits, home health care visits, inpatient emergency room visits, acute hospital stays, stays in skilled nursing facilities, hospice stays, hospital readmissions, and several clinical indicators. These clinical indicators included the presence of poor cognitive functioning, confusion, anxiety or anxiousness, functional dependence, wandering, impaired cognitive awareness, restlessness and agitation, depression, amnesia, aphasia, and the use of any Alzheimer’s disease–related prescription medication.

**Table 4:** Logistic Regression Results for Chronic Condition Warehouse predictive model

Term	Full Model: Beta (95% CI)	Reduced Model: Beta (95% CI)
(Intercept)	-7.9254 (-8.1089, -7.7419)	-8.2513 (-8.4294, -8.0732)
Age	0.0752 (0.0732, 0.0773)	0.0848 (0.0828, 0.0868)
Sex (Female)	-0.0675 (-0.1009, -0.0342)	-0.1094 (-0.1416, -0.0773)
Race, White	0.0801 (0.0279, 0.1323)	-0.0113 (-0.0620, 0.0393)
Race, Other	-0.3169 (-0.3784, -0.2555)	-0.2895 (-0.3494, -0.2295)
Dual Eligibility	-0.327 (-0.3636, -0.2903)	-0.2065 (-0.2418, -0.1713)
Hospital outpatient visits	-0.0006 (-0.0012, 0.0000)	-0.0009 (-0.0015, -0.0004)
Home health visits	0.0039 (0.0035, 0.0044)	0.0055 (0.0050, 0.0060)
ER visits	0.3534 (0.3218, 0.3850)	0.4175 (0.3868, 0.4483)
Acute inpatient hospital stays	-0.1372 (-0.1700, -0.1043)	-0.2516 (-0.2834, -0.2198)
Skilled nursing facility stays	0.2721 (0.2475, 0.2966)	0.2832 (0.2593, 0.3071)
Hospice stays	0.3406 (0.2844, 0.3969)	0.4732 (0.4179, 0.5285)
Readmissions	-0.2011 (-0.2394, -0.1629)	-0.1673 (-0.2043, -0.1303)
Prior functional cognition	0.8905 (0.8430, 0.9381)	-
Confused	0.8966 (0.8446, 0.9486)	1.6909 (1.6528, 1.7291)
Anxious	-0.1546 (-0.1905, -0.1188)	-0.1436 (-0.1780, -0.1093)
Functional dependence	0.5943 (0.5579, 0.6307)	-
Anxiety	-0.2303 (-0.3256, -0.1350)	-0.5100 (-0.6009, -0.4192)
Wandering	0.9415 (0.6652, 1.2178)	1.0533 (0.7818, 1.3249)
Current Cognitive function	-0.7461 (-0.7949, -0.6973)	-
Impulse disorder	1.0703 (0.4777, 1.6629)	1.138 (0.5597, 1.7164)
Amnesia	1.244 (1.0595, 1.4284)	1.3301 (1.1479, 1.5123)
Restlessness and Agitation	0.6774 (0.4669, 0.8878)	0.7469 (0.5418, 0.9520)
Aphasia	0.5042 (0.3755, 0.6328)	0.5613 (0.4359, 0.6867)
Depression	0.7699 (0.7356, 0.8042)	0.6947 (0.6624, 0.7270)
Anxiety medication, consistent use	0.0651 (0.0123, 0.1179)	-
Insomnia medications	1.5953 (0.3973, 2.7932)	-
Dementia medications	1.4344 (1.3244, 1.5444)	-

Legend. Logistic regression coefficients and 95% confidence intervals for variables included in the CCW predictive model. Positive coefficients indicate higher odds of being classified as ADRD-positive, while negative coefficients indicate lower odds. All variables reflect demographic characteristics, healthcare utilization measures, cognitive and functional assessment items, behavioral symptoms, and medication use indicators. The “reduced model” excludes cognitive, functional, behavioral, and medication variables to assess contribution to the model performance. Intercept terms are included for completeness but are not interpreted

Abbreviations: Alzheimer’s Disease and Related Dementias *ADRD*, Chronic Conditions Warehouse *CCW*

**Table 5:** Logistic Regression Results for the ADRD predictive model

Term	Full Model: Beta (95% CI)	Reduced Model: Beta (95% CI)
(Intercept)	-9.0631 (-9.2786, -8.8475)	-8.7244 (-8.9131, -8.5358)
Age	0.0793 (0.0770, 0.0817)	0.0866 (0.0845, 0.0887)
Sex, Female	0.0777 (0.0402, 0.1152)	-0.0755 (-0.1088, -0.0423)
Race, White	-0.0114 (-0.0659, 0.0430)	-0.1915 (-0.2398, -0.1432)
Race, Black	0.2344 (0.1647, 0.3041)	0.2051 (0.1419, 0.2682)
Dual eligibility	0.0873 (0.0452, 0.1295)	0.1672 (0.1307, 0.2038)
Hospital outpatient visits	-0.0031 (-0.0038, -0.0024)	-0.0032 (-0.0038, -0.0026)
Home health visits	0.0055 (0.0049, 0.0060)	0.0061 (0.0056, 0.0066)
ER visits	0.3620 (0.3241, 0.3999)	0.3878 (0.3543, 0.4213)
Acute inpatient hospital stays,	-0.2192 (-0.2585, -0.1799)	-0.3488 (-0.3833, -0.3142)
Skilled nursing facility stays	0.3801 (0.3515, 0.4087)	0.3362 (0.3119, 0.3605)
Hospice stays	0.5343 (0.4683, 0.6002)	0.5769 (0.5216, 0.6321)
Readmissions	-0.1423 (-0.188, -0.0966)	-0.0878 (-0.1264, -0.0493)
Prior functional cognition	1.2285 (1.1735, 1.2834)	-
Confused	1.1843 (1.1233, 1.2453)	2.1305 (2.086, 2.1750)
Anxious	-0.2056 (-0.2465, -0.1648)	-0.2803 (-0.3165, -0.2440)
Functional dependence	0.6953 (0.6540, 0.7366)	-
Anxiety	-3.0088 (-3.2167, -2.8009)	-3.3659 (-3.5554, -3.1764)
Wandering	1.6182 (1.2648, 1.9716)	1.3695 (1.0892, 1.6498)
Current Cognitive functioning	-4.9719 (-5.1178, -4.8259)	-
Amnesia	3.2953 (2.9480, 3.6427)	2.9379 (2.6474, 3.2284)
Executive dysfunction	1.6844 (0.1412, 3.2276)	1.6633 (0.3189, 3.0077)
Restlessness and agitation	0.9097 (0.6652, 1.1542)	0.6248 (0.4318, 0.8178)
Aphasia	0.3067 (0.1678, 0.4457)	0.3844 (0.2623, 0.5065)
Apraxia	1.2267 (0.4867, 1.9667)	0.9129 (0.3564, 1.4695)
Depression	0.3324 (0.2937, 0.3712)	0.0580 (0.0253, 0.0907)
Depression medications, continuous use	0.2352 (0.1462, 0.3242)	-
Dementia medications	1.4464 (1.1597, 1.7330)	-
Dementia medications, continuous use	0.4115 (0.0923, 0.7306)	-

Legend. Logistic regression coefficients and 95% confidence intervals for variables included in the ADRD predictive model. Positive coefficients indicate higher odds of classification as ADRD-highly likely under the ADRD predictive model, while negative coefficients indicate lower odds. The ADRD predictive model incorporates demographic characteristics, healthcare utilization measures, cognitive and functional indicators, behavioral symptoms, and medication use patterns to distinguish ADRD-highly likely cases from ADRD-unlikely and ADRD-possible classifications. The “reduced model” excludes cognitive, functional, behavioral, and medication variables to assess contribution to the model performance. Intercept terms are included for completeness but are not interpreted

Abbreviations: Alzheimer’s Disease and Related Dementias *ADRD*

In addition to these shared variables, the CCW predictive model uniquely included indicators for the presence of impulse disorder, use of any insomnia-related prescription, and repeated prescriptions for anxiety. Conversely, the ADRD predictive model uniquely included indicators for executive dysfunction, apraxia, repeated prescriptions for depression medications, and repeated prescriptions for dementia medications.

### Model Performance

Model performance for predicting ADRD classifications generated by the CCW binary and the ADRD ternary algorithms was evaluated in the independent test dataset. In the training dataset, pooled fit statistics favored the full models over reduced specifications. The CCW predictive model demonstrated lower AIC (101,851 vs. 107,010) and higher adjusted pseudo R² (0.283 vs. 0.247) compared with its reduced counterpart. Similarly, the ADRD predictive model showed substantially improved fit in the full model (AIC: 81,056 vs. 101,311; pseudo R²: 0.432 vs. 0.290). These findings indicate that inclusion of medication and OASIS-derived predictors contributes meaningful explanatory power beyond baseline variables. The two predictive models produced concordant predicted classifications for 89.8% of subjects.

When predicting ADRD status as defined by the CCW binary classification algorithm (ADRD-positive, ADRD-negative), the CCW predictive model achieved an AUC of 0.847, compared with an AUC of 0.813 for the ADRD predictive model. AUC reflects discrimination with respect to the specific outcome and should not be interpreted as a direct comparison of model performance across different outcome definitions. Sensitivity and negative predictive value were higher for the CCW predictive model, whereas specificity and positive predictive value were higher for the ADRD predictive model, indicating a more conservative approach to classification by the ADRD predictive model when evaluated against the CCW defined outcome.

When predicting ADRD status as defined by the ADRD ternary classification algorithm (ADRD-highly likely, ADRD-possible, ADRD-unlikely), the ADRD predictive model achieved a higher AUC (0.906) compared to the CCW predictive model (0.847). In this context, the ADRD predictive model demonstrated higher specificity and positive predictive value, while sensitivity and negative predictive value were similar across models.

**Fig. 2:**
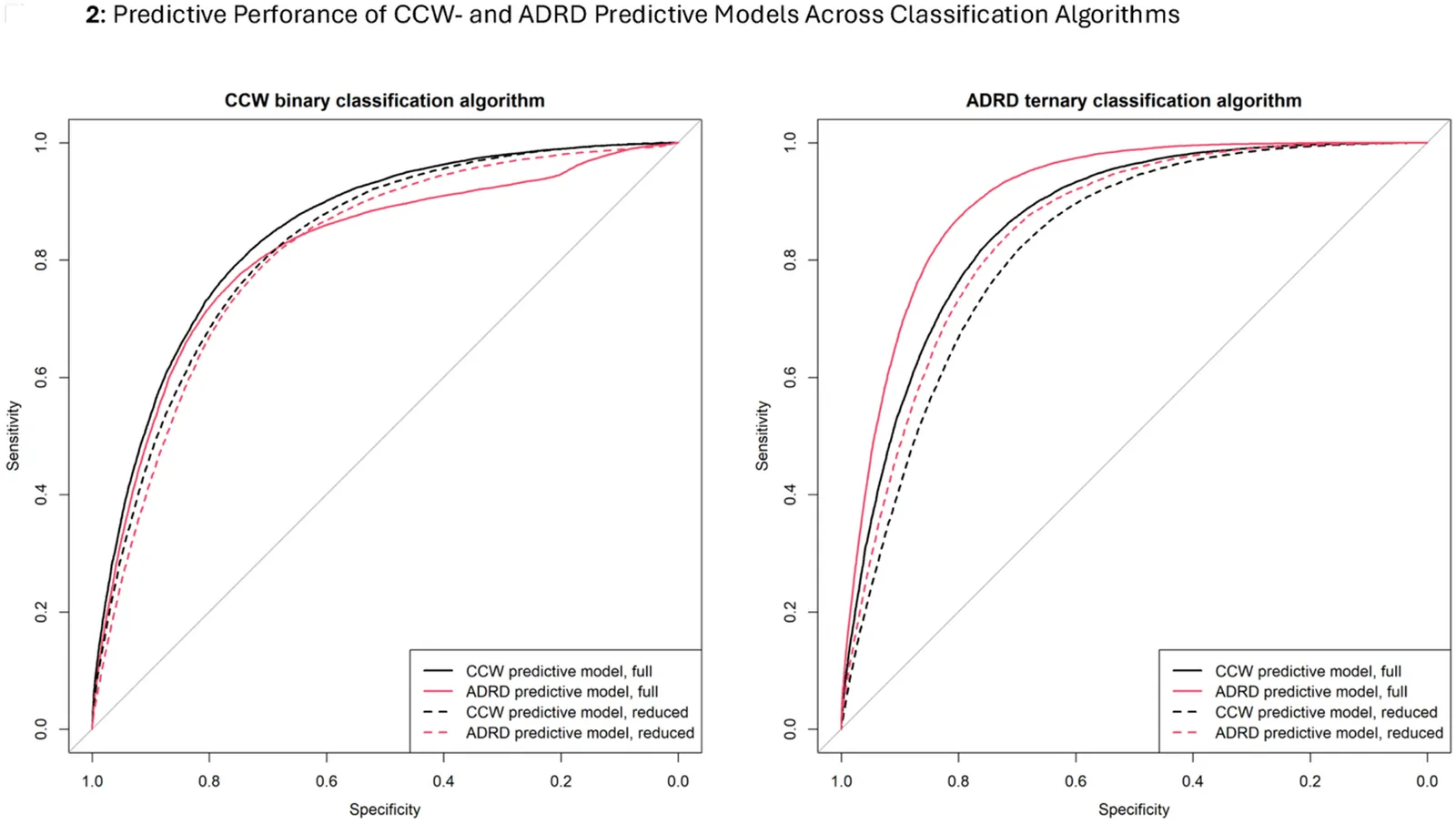
Receiver operating characteristic (ROC) curves for CCW and ADRD predictive models by classification algorithm. (Legend) Receiver operating characteristic (ROC) curves for predictive models trained to identify ADRD status as defined by two classification algorithms. In the left panel, all models predict ADRD status as defined by the CCW binary classification algorithm. In the right panel, all models predict ADRD status as defined by the ADRD ternary classification algorithm. Black lines represent CCW predictive models and red lines represent ADRD predictive models. Solid and dashed lines denote full and reduced models, respectively, with reduced models removing medication and OASIS variables. Importantly, differences between curves within each panel reflect variation in predictive model specification rather than differences in underlying classification algorithm, as the outcome is held constant within each panel. Higher AUCs for models aligned with their corresponding classification algorithm reflect stronger structural alignment with algorithm-defined labels rather than differences in underlying disease status

Importantly, within each evaluation, both predictive models were assessed against the same classification algorithm defined outcome. Thus, differences in AUC reflect variation in model specification and alignment with the underlying classification algorithm definition, rather than differences in the algorithm itself. In both evaluations, predictive models demonstrated strong discrimination (AUCs > 0.80), with higher performance observed when the predictive model was aligned with its corresponding classification algorithm. Performance attenuation in reduced models suggests that these variables contribute meaningful predictive signal beyond baseline utilization measures, especially OASIS-derived predictors. Performance metrics are summarized in Table 6, and ROC curves for both outcomes are presented in Fig. 2, stratified by classification algorithm. Model performance was further evaluated using calibration metrics and optimized classification thresholds. These results are summarized in (Supplemental Table 4). Optimized classification thresholds varied across models and outcomes, reflecting differences in outcome prevalence and intended application. Ablation analyses demonstrated that exclusion of medication and OASIS variables resulted in modest attenuation of effect sizes but did not substantially alter overall model structure (Supplemental Tables 2 and 3).

**Table 6:** Performance of CCW and ADRD Predictive Models Across Classification Algorithms

	CCW binary classification algorithm	ADRD ternary classification algorithm
Full Models		
CCW predictive model		
AUC (95% CI)	0.8473 (0.8442, 0.8473)	0.8628 (0.8599, 0.8628)
Optimal Threshold	0.600	0.625
Sensitivity (95% CI)	0.7361 (0.7312, 0.7409)	0.7605 (0.7554, 0.7655)
Specificity (95% CI)	0.8011 (0.7965, 0.8057)	0.8022 (0.7978, 0.8065)
PPV (95% CI)	0.8018 (0.7972, 0.8064)	0.7666 (0.7616, 0.7716)
NPV (95% CI)	0.7352 (0.7303, 0.7401)	0.7967 (0.7923, 0.8011)
ADRD predictive model		
AUC (95% CI)	0.8132 (0.8097, 0.8132)	0.906 (0.9037, 0.906)
Optimal Threshold	0.555	0.480
Sensitivity (95% CI)	0.7184 (0.7135, 0.7234)	0.8709 (0.8669, 0.8748)
Specificity (95% CI)	0.8004 (0.7958, 0.8051)	0.8002 (0.7959, 0.8046)
PPV (95% CI)	0.7974 (0.7927, 0.8021)	0.7883 (0.7838, 0.7929)
NPV (95% CI)	0.7223 (0.7173, 0.7272)	0.8788 (0.8751, 0.8826)
Agreement between Models	0.9042 (0.9019, 0.9066)	0.8759 (0.9733, 0.8785)
AUC for concordant predictions	0.9074 (0.9049, 0.9099)	0.9253 (0.9231, 0.9276)
AUC for discordant predictions	0.6099 (0.5982, 0.6217)	0.8145 (0.8062, 0.8229)
Reduced Models (exclude medication use and OASIS)		
CCW predictive model		
AUC (95% CI)	0.8252 (0.8219, 0.8252)	0.8231 (0.8198, 0.8231)
Optimal Threshold	0.625	0.660
Sensitivity (95% CI)	0.6824 (0.6772, 0.6875)	0.6659 (0.6604, 0.6715)
Specificity (95% CI)	0.8005 (0.7958, 0.8051)	0.8012 (0.7968, 0.8055)
PPV (95% CI)	0.789 (0.7841, 0.7938)	0.7411 (0.7356, 0.7465)
NPV (95% CI)	0.6974 (0.6925, 0.7024)	0.7373 (0.7327, 0.7419)
AUC (95% CI)	0.8102 (0.8067, 0.8102)	0.8477 (0.8447, 0.8477)
Optimal Threshold	0.565	0.565
Sensitivity (95% CI)	0.6692 (0.664, 0.6744)	0.7324 (0.7272, 0.7377)
Specificity (95% CI)	0.8003 (0.7956, 0.8049)	0.8007 (0.7964, 0.8051)
PPV (95% CI)	0.7855 (0.7806, 0.7905)	0.7585 (0.7533, 0.7636)
NPV (95% CI)	0.6888 (0.6838, 0.6937)	0.7779 (0.7734, 0.7824)
Agreement between Models	0.9035 (0.9012, 0.9059)	0.8940 (0.8916, 0.8965)
AUC for concordant predictions	0.8752 (0.8722, 0.8782)	0.8838 (0.8809, 0.8867)
AUC for discordant predictions	0.5441 (0.5321, 0.5562)	0.6885 (0.6775, 0.6995)

Legend. Performance metrics, including area under the curve (AUC), sensitivity, specificity, positive predictive value (PPV), and negative predictive value (NPV), for the CCW and novel ADRD models. Metrics are reported for each model’s ability to predict both CCW and novel ADRD algorithm classifications. Additional measures include overall agreement between models and AUC for concordant and discordant predictions, providing insight into model consistency and discriminatory performance. Metrics reflect each model’s performance when predicting ADRD status defined by each classification algorithm. Comparisons of AUC should be made within the same outcome column (CCW or ADRD), not across columns. AUC reflects discrimination for the specified outcome. Comparisons across outcomes should not be interpreted as direct measures of relative model performance. Calibration metrics and optimized classification thresholds are reported in Supplemental Table 3

Abbreviations: Alzheimer’s Disease and Related Dementias *ADRD*, Chronic Conditions Warehouse *CCW*

citation for pseudo=R2: McFadden, Daniel (1972). “Conditional logit analysis of qualitative choice behavior”. Working Paper Np. 199/BART 10: 23

### Classification and prediction of beneficiaries with ADRD-possible status

Among the 63,358 beneficiaries classified as ADRD-possible by the ADRD ternary classification algorithm and with complete data, the CCW binary classification algorithm classified 32,436 as ADRD-negative and 32,902 as ADRD-positive. When applied to this group, the CCW predictive model predicted 44.2% (28,904) as ADRD-negative and 55.8% (36,434) as ADRD-positive, while the ADRD predictive model predicted 61.8% (40,371) as ADRD-negative and 38.2% (24,967) as ADRD-positive.

Among those classified as ADRD-negative by the CCW binary classification algorithm, the ADRD predictive model more closely aligned with the ADRD-negative labels defined by the CCW binary classification algorithm (22,558 vs. 18,483) and generated fewer label disagreements (9,878 vs. 13,953) than the CCW predictive model. Conversely, among those classified as ADRD-positive by the CCW binary classification algorithm, the CCW predictive model showed greater agreement with the CCW binary algorithm-defined ADRD positive labels (22,481 vs. 15,089) and fewer false negatives (10,421 vs. 17,813) than the ADRD predictive model.

**Fig. 3:**
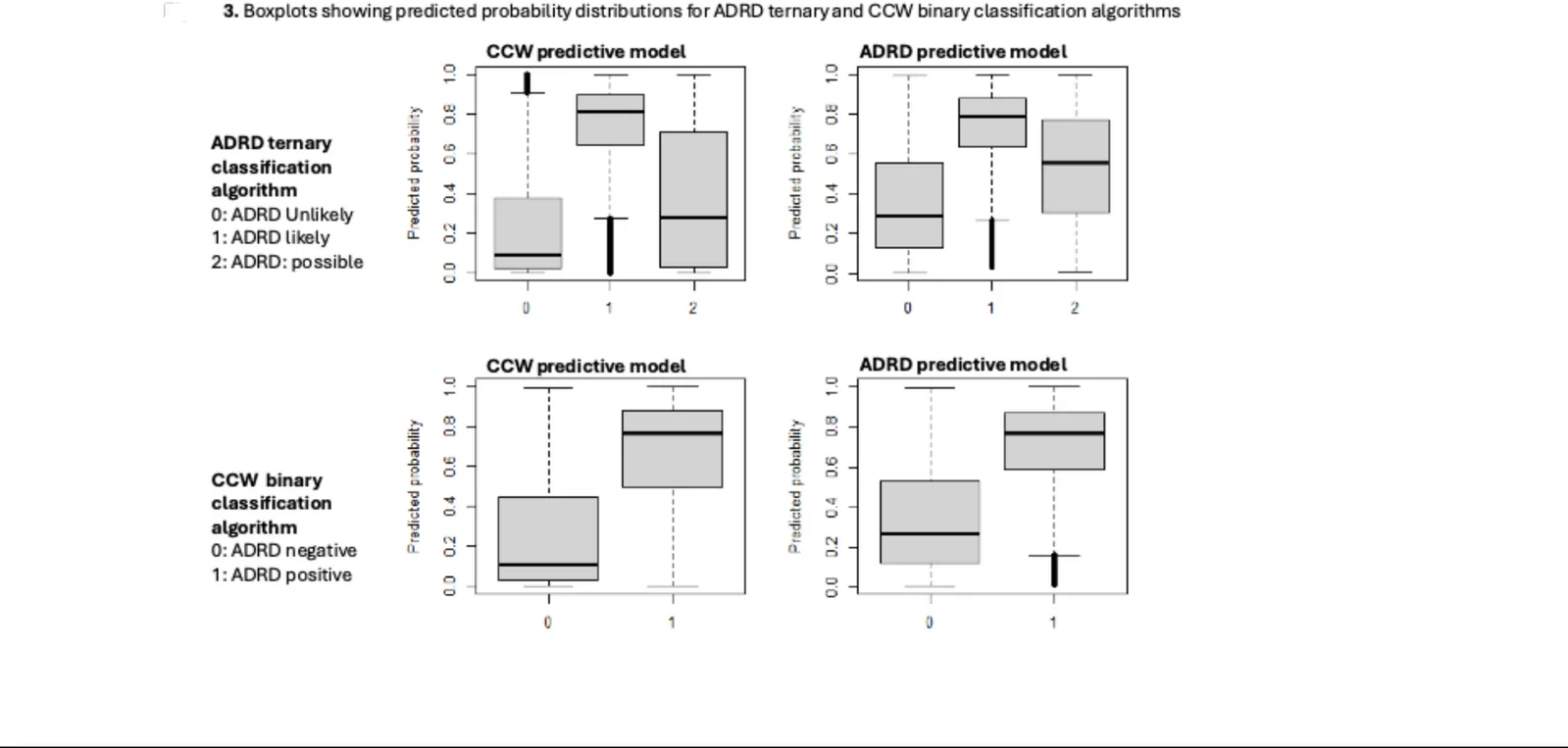
Predictive Probability Distributions by Model and ADRD Classification Group. (Legend) Distribution of predicted probabilities by model and classification group. The top row shows predicted probabilities for the ADRD ternary classification algorithm and the second row shows predicted probabilities for the Chronic Conditions Warehouse (CCW) binary classification algorithm. Columns indicate predictions from the CCW predictive model (column 1) and the ADRD predictive model (column 2). Overall, the models produced concordant classifications for 80.6% of beneficiaries with ADRD-possible status. Separation of distributions illustrates differences in how each model classifies individuals with ADRD-possible status, with the ADRD predictive model showing greater concentration toward lower predictive probabilities. (Abbreviations: ADRD = Alzheimer’s disease and related dementias; CCW = Chronic Conditions Warehouse)

Predicted probability distributions by model and classification algorithm group are presented in Fig. 3 illustrating greater separation of predicted probabilities under the ADRD predictive model, particularly among individuals classified as ADRD-negative. Detailed performance results are shown in Table 7.

**Table 7:** Predicted ADRD Classification Among Beneficiaries with ADRD-Possible Status

CCW classification algorithm	Prediction	CCW Predictive Model	ADRD Predictive Model
ADRD-negative	ADRD-negative/ADRD-unlikely	18,483 of 32,436	27,507 of 32,436
ADRD-negative	ADRD-positive/ ADRD-Highly Likely	13,953 of 32,436	9,878 of 32,436
ADRD-positive	ADRD-negative/ADRD-unlikely	10,421 of 32,902	17,813 of 32,902
ADRD-positive	ADRD-positive/ ADRD-Highly Likely	22,481 of 32,902	15,089 of 32,902

Legend. Predicted ADRD classifications for ADRD-possible patients are shown relative to CCW classification. Counts represent model-predicted classifications relative to CCW binary classification algorithm defined ADRD status among beneficiaries classified as ADRD-possible by the ADRD ternary classification algorithm. This comparison highlights differences in model predictions for participants with ADRD- possible status

Abbreviations: Alzheimer’s Disease and Related Dementias *ADRD*, Chronic Conditions Warehouse *CCW*

## Discussion

This study evaluated whether NPS codes and medication billing data could improve the prediction of ADRD classifications generated by the CCW binary classification algorithm and by an ADRD ternary classification algorithm. Importantly, these variables were not added to the classification algorithms themselves but were used as predictors in regression models assessing their ability to explain algorithmic classifications. Although elastic net regression reduces overfitting relative to stepwise approaches, variable selection remains dependent on the underlying structure and may yield different parameterizations across models, as reflected in Supplemental Tables 2 and 3. We also did not formally compare AUCs across different outcome definitions, as these represent distinct classification targets rather than a shared ground truth. We found that models integrating these data sources, particularly when trained on the ADRD ternary classification algorithm, performed comparably or better in reproducing ADRD classifications generated by the CCW binary classification algorithm. The ADRD predictive model showed the greatest advantage when applied to beneficiaries with ADRD-possible status, classifying a larger proportion as ADRD-negative compared with the CCW predictive model and suggesting greater specificity in distinguishing algorithm-defined ADRD-positive from ADRD-negative classifications among beneficiaries with similar documented clinical characteristics. In the ADRD-possible subgroup, which was excluded from model training, the ADRD predictive model’s performance highlights the potential to enhance classification validity for future research. Notably, the ADRD-possible group does not represent a simple intermediate category between ADRD-highly likely and ADRD-unlikely classifications, but rather a heterogeneous group defined by diagnostic uncertainty, partial coding, or exclusionary clinical conditions.

Among beneficiaries with a definitive ADRD status (i.e., not ADRD-possible), the CCW and novel ADRD predictive models exhibited strong discrimination (AUCs > 0.81). However, primary performance measures diverged. The CCW predictive model favored sensitivity, capturing a broader set of potential cases; while the ADRD predictive model demonstrated higher specificity and positive predictive value, reflecting a more conservative, clinically informed classification strategy. This difference is consistent with the design of the ADRD ternary classification algorithm, which uses a refined ICD-10 ADRD diagnosis list and exclusionary criteria aligned with clinical diagnostic standards. The inclusion of NPS ICD-10 codes and medication data further improved prediction among ambiguous cases (Novel ADRD algorithm, ADRD-possible), indicating that treatment and behavioral symptom profiles may provide valuable signals for predicting algorithm-defined ADRD classifications beyond diagnostic codes alone. Sensitivity analyses excluding medication and OASIS-derived variables yielded similar patterns of association, suggesting that model performance was not solely driven by predictors closely tied to ADRD documentation processes (Supplemental Tables 2 and 3).

Our findings contribute to a growing body of literature identifying limitations in existing claims-based ADRD identification strategies. Prior work, including that of Gianattasio et al. and McCarthy et al., has highlighted variability in algorithm performance and the risk of false positives when single-claim definitions such as the CCW binary classification algorithm are used [15, 23]. By integrating ICD-10 codes for NPS and medication billing data, our results demonstrate that these additional data sources improve the ability to predict ADRD classifications generated by both the CCW binary and ADRD ternary classification algorithms, particularly for beneficiaries with ambiguous (ADRD-possible) status.

The ADRD ternary classification algorithm incorporates clinically informed refinements, including a restricted ICD-10 code set, repeated diagnostic claims, and exclusionary conditions intended to reduce misclassification arising from transient or uncertain diagnostic coding. In this study, models trained on the ADRD ternary classification algorithm demonstrated stronger performance when evaluated against the ADRD outcome and comparable or improved performance in predicting CCW outcomes, suggesting stronger alignment with the structure of the ADRD ternary classification algorithm rather than improved identification of true underlying disease.

However, it is important to emphasize that these findings reflect agreement with algorithm-defined labels rather than validation against a clinical gold standard. Accordingly, higher predictive performance for the ADRD ternary classification algorithm should be interpreted as evidence that this classification algorithm may be more internally consistent and more strongly structured in relation to available claims-based predictors, rather than as proof of superior clinical diagnostic accuracy. The observed differences in model performance therefore reflect variation in how well each classification algorithm is captured by structural administrative and clinical data, rather than definitive differences in true disease status. Future studies using clinically adjudicated outcomes are necessary to confirm validity and to assess the potential for reducing misclassification-related biases, including those that may disproportionately affect racial and ethnic groups; stratified analyses will be important to evaluate these effects [24, 25]. 

The ADRD ternary classification algorithm showed the greatest divergence from the CCW binary classification algorithm among beneficiaries with ADRD-possible status, where the incorporation of neuropsychiatric symptom codes and medication data improved separation between algorithm-defined categories. This finding suggests that patterns and behavioral symptom indicators may provide additional structure beyond diagnosis codes alone, particularly in clinically ambiguous cases.

This study also demonstrates the methodological value of separating rule-based phenotyping (the CCW binary and ADRD ternary classification algorithms) from model-based performance using additional real-world data. Whereas the algorithms generate deterministic labels, the predictive models leverage probabilistic learning to identify subtle patterns in utilization and symptom presentation. Incorporating NPS and medication data, particularly from Medicare Part D, may capture clinical nuances that are missed by diagnostic coding alone and thus improve ADRD identification in administrative datasets.

Finally, the use of OASIS home health data underscores the potential of integrating behavioral, cognitive, and functional indicators into claims-based research. Nearly one-third of community-dwelling older-adults with ADRD receive HH services, yet these data are rarely linked in large-scale analysis [26–28] making this an underutilized yet rich source of data for dementia research. Future studies should consider this data source when developing or validating ADRD classification models.

## Limitations

Several limitations merit consideration. First, this study lacks a clinical gold standard for ADRD diagnosis. Although our ADRD ternary classification algorithm was developed to improve upon existing classifications, it is based on administrative and behavioral data rather than direct cognitive testing or clinician adjudication. Prior studies have shown that even robust claims-based classification algorithms exhibit substantial misclassification when compared with clinical assessments. Accordingly, higher model performance reflects stronger agreement with algorithm-defined labels rather than improved detection of clinically confirmed ADRD.

Second, because many predictors (e.g., dementia medications, neuropsychiatric symptom codes, and cognitive assessments) are derived from the same clinical care processes that inform ICD-10 dementia diagnoses, the models may partially reflect patterns of healthcare utilization and documentation in addition to underlying disease. However, sensitivity analyses excluding these variable groups yielded similar model performance, suggesting that findings are not solely driven by such factors (Supplemental Tables 2–4).

Third, while multiple imputation and rigorous model selection strategies were employed, missing data in cognitive and psychiatric variables may have introduced bias, particularly among marginalized populations for whom assessment completeness may vary. Although elastic net reduces overfitting relative to stepwise approaches, variable selection remains dependent on the underlying data structure and may yield different parameterizations across models. Fourth, the study cohort was limited to fee-for-service Medicare beneficiaries receiving home health services. Results may not generalize to Medicare Advantage populations or to settings where structured cognitive, behavioral, and functional data are not readily available. Similarly, because HH eligibility requires individuals to be homebound, this cohort likely represents a more clinically impaired population than the broader community-dwelling older adult population. We did not perform formal bias adjustment (e.g., propensity-based methods), which may limit generalizability of these findings to other populations.

Fifth, although the ADRD ternary classification algorithm includes exclusionary criteria to improve specificity of classification, this may inadvertently exclude individuals with comorbid psychiatric or neurologic conditions who do have ADRD. This trade-off highlights an inherent tension between algorithmic precision and clinical inclusivity, particularly for individuals with overlapping symptom profiles. Sixth, the predictive models relied on cross-sectional data with a 1-year look-back period, which may limit their ability to capture incident ADRD or longitudinal disease progression.

Finally, while we examined agreement and performance between models, formal validation against clinically adjudicated diagnoses remains necessary. Additionally, ICD-10 coding practices may vary across providers and settings, which could affect model performance when applied to other publications. Importantly, NPS ICD-10 codes and medication data were used solely as predictors in regression models and were not incorporated into the pre-defined classification algorithms; their predictive value should be confirmed in future studies.

## Conclusions

Predictive models incorporating NPS codes and medication data improved the characterization of algorithm-defined ADRD status, particularly among individuals with uncertain classification. Models aligned with the ADRD ternary classification algorithm demonstrated higher specificity and fewer discordant classifications, reflecting a more conservative approach to phenotype definition. While these findings do not establish clinical diagnostic validity, they highlight the value of integrating behavioral, cognitive, and treatment-related data to refine administrative phenotyping strategies. Future work should validate these approaches against clinically adjudicated diagnoses and assess their performance across diverse populations.

## Supplementary information

Supplementary Material 1.

Supplementary Material 2.

Supplementary Material 3.

Supplementary Material 4.

Supplementary Material 5.

## Data Availability

The data that support the findings of this study are available from the Centers for Medicare and Medicaid Services but restrictions apply to the availability of these data, which were used under license for the current study, and so are not publicly available. Data can be obtained directly from the Centers for Medicare and Medicaid Services.

## Abbreviations

ADRD: Alzheimer’s Disease and Related Dementias
ICD-10: International Classification of Diseases, Tenth Revision
CCW: Centers for Medicare & Medicaid Services Chronic Conditions Warehouse
NPS: Neuropsychiatric symptoms
HH: Home Health
MedPAR: Medicare Provider Analysis and Review
FFS: Fee-for-Service
MBSF: Medicare Beneficiary Summary Files
OASIS: Outcomes and Assessment Information Set
PPV: Positive Predictive Value
NPV: Negative Predictive Value
AUC: Area Under the Receiver Operating Characteristic Curve
NRI: Net Reclassification Improvement
MICE: Multiple Imputation by Chained Equations
SAENET: Stacked Adaptive Elastic Net Regression (R package)
χ²: Chi-square
