# Supplementary material for: Methodological advances in claims-based dementia algorithms: integrating medication and clinical data for medicare populations
Source: BMC Med Res Methodol. 2026 Jun 25;26:207. doi: 10.1186/s12874-026-02929-7 (PMC13573462; doi:10.1186/s12874-026-02929-7)
Supplement: Supplementary file 1 [file 12874_2026_2929_MOESM1_ESM.docx]

| **ALZHEIMER'S DEMENTIA BY CCW** |  |  |  |  |
| --- | --- | --- | --- | --- |
|  |  |  |  |  |
|  | **Full Model: Beta (95% CI)** | **No Medication Model: Beta (95% CI)** | **No OASIS Model: Beta (95% CI)** | **No Medication or OASIS Model: Beta (95% CI)** |
| Intercept | -7.9254  (-8.1089, -7.7419) | -7.915  (-8.098, -7.7321) | -8.2653  (-8.4442, -8.0865) | -8.2513  (-8.4294, -8.0732) |
| Age, 1 year increase | 0.0752  (0.0732, 0.0773) | 0.0751  (0.0730, 0.0771) | 0.0848  (0.0828, 0.0869) | 0.0848  (0.0828, 0.0868) |
| Sex, Female vs. Male | -0.0675  (-0.1009, -0.0342) | -0.0656  (-0.0987, -0.0324) | -0.1084  (-0.1408, -0.0760) | -0.1094  (-0.1416, -0.0773) |
| Race, White vs. Black | 0.0801  (0.0279, 0.1323) | 0.0992  (0.0472, 0.1512) | -0.0255  (-0.0764, 0.0255) | -0.0113  (-0.0620, 0.0393) |
| Race, Other vs. Black | -0.3169  (-0.3784, -0.2555) | -0.2868  (-0.3479, -0.2257) | -0.3194  (-0.3798, -0.2591) | -0.2895  (-0.3494, -0.2295) |
| Dual eligibility, Yes vs. No | -0.327  (-0.3636, -0.2903) | -0.3168  (-0.3533, -0.2803) | -0.2178  (-0.2533, -0.1824) | -0.2065  (-0.2418, -0.1713) |
| Hospital out patient visits, increase of 1 visit | -0.0006  (-0.0012, 0.0000) | -7e-04  (-0.0013, -0.0001) | -0.0008  (-0.0013, -0.0002) | -0.0009  (-0.0015, -0.0004) |
| Home health visits, increase of 1 visit | 0.0039  (0.0035, 0.0044) | 0.0040  (0.0035, 0.0045) | 0.0054  (0.0049, 0.0059) | 0.0055  (0.0050, 0.0060) |
| In patient ER visits, increase of 1 visit | 0.3534  (0.3218, 0.3850) | 0.3589  (0.3273, 0.3904) | 0.4108  (0.3799, 0.4416) | 0.4175  (0.3868, 0.4483) |
| Acute in patient hospital stays, increase of 1 stay | -0.1372  (-0.1700, -0.1043) | -0.1473  (-0.1801, -0.1146) | -0.2394  (-0.2713, -0.2074) | -0.2516  (-0.2834, -0.2198) |
| Skilled nursing facility stays, increase of 1 stay | 0.2721  (0.2475, 0.2966) | 0.2681  (0.2436, 0.2926) | 0.2864  0.2624, 0.3103) | 0.2832  (0.2593, 0.3071) |
| Hospice stays, increase of 1 stay | 0.3406  (0.2844, 0.3969) | 0.3446  (0.2885, 0.4007) | 0.4681  (0.4126, 0.5236) | 0.4732  (0.4179, 0.5285) |
| Readmissions, increase of 1 readmission | -0.2011  (-0.2394, -0.1629) | -0.1969  (-0.235, -0.1588) | -0.1721  (-0.2092, -0.1349) | -0.1673  (-0.2043, -0.1303) |
| Current Cognitive functioning impaired, Yes vs. No | 0.8905  (0.8430, 0.9381) | 0.9123  (0.8650, 0.9595) | - | - |
| Confused, Yes vs. No | 0.8966  (0.8446, 0.9486) | 0.9111  (0.8596, 0.9627) | 1.656  (1.6174, 1.6945) | 1.6909  (1.6528, 1.7291) |
| Anxious, Yes vs. No | -0.1546  (-0.1905, -0.1188) | -0.1542  (-0.1898, -0.1187) | -0.1406  (-0.1753, -0.1059) | -0.1436  (-0.1780, -0.1093) |
| Functional dependence, Yes vs. No | 0.5943  (0.5579, 0.6307) | 0.6039  (0.5674, 0.6404) | - | - |
| Anxiety, Yes vs. No | -0.2303  (-0.3256, -0.1350) | -0.2309  (-0.3257, -0.1362) | -0.4887  (-0.5803, -0.3971) | -0.5100  (-0.6009, -0.4192) |
| Wandering, Yes vs. No | 0.9415  (0.6652, 1.2178) | 0.9481  (0.6738, 1.2225) | 1.0507  (0.7774, 1.3241) | 1.0533  (0.7818, 1.3249) |
| Cognitive functional awareness impaired, Yes vs. No | -0.7461 (-0.7949, -0.6973) | -0.7521  (-0.8003, -0.7039) | - | - |
| Impulse disorder, Yes vs. No | 1.0703  (0.4777, 1.6629) | 1.0719  (0.4841, 1.6597) | 1.1239  (0.5402, 1.7076) | 1.138  (0.5597, 1.7164) |
| Amnesia, Yes vs. No | 1.244  (1.0595, 1.4284) | 1.2439  (1.0594, 1.4285) | 1.3296  (1.1473, 1.5119) | 1.3301  (1.1479, 1.5123) |
| Restlessness and agitation, Yes vs. No | 0.6774  (0.4669, 0.8878) | 0.7033  (0.4933, 0.9132) | 0.7241  (0.5180, 0.9303) | 0.7469  (0.5418, 0.9520) |
| Aphasia, Yes vs. No | 0.5042  (0.3755, 0.6328) | 0.5001  (0.3717, 0.6284) | 0.5652  (0.4395, 0.6910) | 0.5613  (0.4359, 0.6867) |
| Depression, Yes vs. No | 0.7699  (0.7356, 0.8042) | 0.7831  (0.749, 0.8172) | 0.6879  (0.6552, 0.7205) | 0.6947  (0.6624, 0.7270) |
| Consistent anxiety medications, Yes vs. No | 0.0651  (0.0123, 0.1179) | - | -0.0420  (-0.0925, 0.0086) | - |
| Any insomnia medications, Yes vs. No | 1.5953  (0.3973, 2.7932) | - | 1.6391  (0.4882, 2.7900) | - |
| Any dementia medications, Yes vs. No | 1.4344  (1.3244, 1.5444) | - | 1.5598  (1.4517, 1.6679) | - |

Intercept terms are included for completeness but are not interpreted. Coefficients are presented to illustrate relative effect size and direction
