# Supplementary material for: Methodological advances in claims-based dementia algorithms: integrating medication and clinical data for medicare populations
Source: BMC Med Res Methodol. 2026 Jun 25;26:207. doi: 10.1186/s12874-026-02929-7 (PMC13573462; doi:10.1186/s12874-026-02929-7)
Supplement: Supplementary file 2 [file 12874_2026_2929_MOESM2_ESM.docx]

| **Supplemental Table 1a.** International Classification of Disease Tenth revision (ICD-10) codes contained in both algorithms | | | |
| --- | --- | --- | --- |
| **ICD- Codes** | **Description** | **CCW Binary Classification algorithm** | **ADRD Ternary Classification Algorithm** |
| F01.50 | Vascular dementia without behavioral disturbance | X | X |
| F01.51 | Vascular dementia with behavioral disturbance | X | X |
| F02.80 | Dementia in other diseases classified elsewhere without behavioral disturbance | X | X |
| F02.81 | Dementia in other diseases classified elsewhere with behavioral disturbances | X | X |
| F03.90 | Unspecified dementia without behavioral disturbance | X | X |
| F03.91 | Unspecified dementia with behavioral disturbance | X | X |
| F04 | Amnestic disorder due to known physiological condition | X |  |
| F05 | Delirium due to known physiological condition | X |  |
| F06.1 | Catatonic disorder due to known physiological condition | X |  |
| F06.8 | Other specified mental disorders due to known physiological conditions | X |  |
| G13.8 | Systemic atrophy primarily affecting central nervous system in other diseases classified elsewhere | X |  |
| G30.0 | Alzheimer’s disease with early onset | X | X |
| G30.1 | Alzheimer’s disease with late onset | X | X |
| G30.8 | Other Alzheimer’s disease | X | X |
| G30.9 | Alzheimer’s disease, unspecified | X | X |
| G31.1 | Senile degeneration of brain, not elsewhere classified | X |  |
| G31.2 | Degeneration of nervous system due to alcohol | X |  |
| G31.01 | Pick’s disease | X | X |
| G31.09 | Other frontotemporal dementia | X | X |
| G94 | Other disorders of brain in diseases classified elsewhere | X |  |
| R41.81 | Age-related cognitive decline | X | X |
| R54 | Age-related physical debility | X |  |

| **Supplement Table 1b: ADRD Ternary Classification Algorithm Exclusion Criteria** | | |
| --- | --- | --- |
| **Exclusion Criteria** | **ICD Code** | **Description** |
| Delirium | F05 | Delirium due to known physiological condition |
|  | R41.0 | Disorientation, unspecified |
|  | R41.82 | Altered mental state, unspecified |
| Cerebrovascular Accident | I63.9 | Cerebral infarction, unspecified |
|  | I67.81 | Acute cerebrovascular insufficiency |
|  | I69.91 | Cognitive deficits following unspecified cerebrovascular disease |
|  | I63.30 | Cerebral infarct due to thrombosis of unspecified cerebral artery |
|  | I63.31 | Cerebral infarct due to thrombosis of middle cerebral artery |
|  | I63.32 | Cerebral infarct due to thrombosis of anterior cerebral artery |
|  | I63.33 | Cerebral infarct due to thrombosis of posterior cerebral artery |
|  | I63.39 | Cerebral infarct due to thrombosis of other cerebral artery |
|  | I63.41 | Cerebral infarct due to embolism of middle cerebral artery |
|  | I63.42 | Cerebral infarct due to embolism of anterior cerebral artery |
|  | I63.43 | Cerebral infarct due to embolism of posterior cerebral artery |
| Neurological disease | G31.83 | Dementia with Lewy bodies |
|  | G10.0 | Frontotemporal dementia |
|  | G10 | Huntington’s disease |
|  | G20 | Parkinson’s disease |
|  | G21.9 | Secondary parkinsonism, unspecified |
|  | G21.0 | Secondary parkinsonism, unspecified |
|  | G21.19 | Other drug induced parkinsons |
|  | G21.4 | Vascular parkinsonism |
|  | G90.3 | Multi-system degeneration of autonomic nervous system |
|  | G21.8 | Other secondary parkinsonism |
|  | G12.21 | Amyotrophic lateral sclerosis |
|  | G35 | Multiple sclerosis |
| Psychiatric Condition | F10 | Alcohol related disorders |
|  | F11 | Opioid related disorders |
|  | F12 | Cannabis related disorders |
|  | F13 | Sedative, hypnotic, or anxiolytic related disorders |
|  | F14 | Cocaine related disorders |
|  | F15 | Other stimulant related disorders |
|  | F16 | Hallucinogen related disorders |
|  | F17 | Nicotine dependence |
|  | F18 | Inhalant related disorders |
|  | F19 | Other psychoactive substance related disorders |
|  | F20 | Schizophrenia |
|  | F21 | Schizotypal disorder |
|  | F22 | Delusional disorders |
|  | F23 | Brief psychotic disorder |
|  | F24 | Shared psychotic disorder |
|  | F25 | Schizoaffective disorders |
|  | F28 | Other psychotic disorder not due to a substance or known physiological condition |
|  | F29 | Unspecified psychosis not due to a substance or known physiological condition |
|  | F31 | Bipolar disorder |
|  | F34 | Persistent mood [affective] disorders |
|  | F39 | Unspecified mood [affective] disorder |
|  | F40 | Phobic anxiety disorders |
|  | F41 | Other anxiety disorders |
|  | F42 | Obsessive-compulsive disorder |
|  | F43 | Reaction to severe stress, and adjustment disorders |
|  | F44 | Dissociative and conversion disorders |
|  | F45 | Somatoform disorders |
|  | F48 | Other nonpsychotic mental disorders |
|  | F50 | Eating disorders |
|  | F51 | Sleep disorders not due to a substance or known physiological condition |
|  | F52 | Sexual dysfunction not due to a substance or known physiological condition |
|  | F53 | Mental and behavioral disorders associated with the puerperium, not elsewhere classified |
|  | F54 | Psychological and behavioral factors associated with disorders or diseases classified elsewhere |
|  | F55 | Abuse of non-psychoactive substances |
|  | F59 | Unspecified behavioral syndromes associated with physiological disturbances and physical factors |
|  | F60 | Specific personality disorders |
|  | F63 | Impulse disorders |
|  | F64 | Gender identity disorders |
|  | F65 | Paraphilias |
|  | F66 | Other sexual disorders |
|  | F68 | Other disorders of adult personality and behavior |
|  | F69 | Unspecified disorder of adult personality and behavior |
|  | F99 | Mental disorder, not otherwise specified |
| Mild Cognitive Impairment | G31.84 | Mild Cognitive Impairment |
|  | R41 | Other symptoms and signs involving cognitive functions and awareness |
|  | R41.8 | Other symptoms and signs involving cognitive functions and awareness |
|  | R41.841 | Cognitive communication deficit |
