# Supplementary material for: Methodological advances in claims-based dementia algorithms: integrating medication and clinical data for medicare populations
Source: BMC Med Res Methodol. 2026 Jun 25;26:207. doi: 10.1186/s12874-026-02929-7 (PMC13573462; doi:10.1186/s12874-026-02929-7)
Supplement: Supplementary file 3 [file 12874_2026_2929_MOESM3_ESM.docx]

| **ADRD OUTCOME** |  |  |  |  |
| --- | --- | --- | --- | --- |
| **SUPP TABLE 2** |  |  |  |  |
|  | **Full Model:**  **Beta (95% CI)** | **No Medication Model: Beta (95% CI)** | **No OASIS Model:**  **Beta (95% CI)** | **No Medication or OASIS Model:**  **Beta (95% CI)** |
| Intercept | -9.0631  (-9.2786, -8.8475) | -8.9977  (-9.2119, -8.7835) | -8.784  (-8.974, -8.5941) | -8.7244  (-8.9131, -8.5358) |
| Age, 1 year increase | 0.0793  (0.0770, 0.0817) | 0.0789  (0.0765, 0.0812) | 0.0869  (0.0848, 0.0890) | 0.0866  (0.0845, 0.0887) |
| Sex, Female vs. Male | 0.0777  (0.0402, 0.1152) | 0.0798  (0.0425, 0.1170) | -0.0764  (-0.1099, -0.0429) | -0.0755  (-0.1088, -0.0423) |
| Race, White vs. Other | -0.0114  (-0.0659, 0.0430) | -0.0156  (-0.0697, 0.0386) | -0.1865  (-0.2352, -0.1378) | -0.1915  (-0.2398, -0.1432) |
| Race, Black vs. Other | 0.2344  (0.1647, 0.3041) | 0.2045  (0.1351, 0.2739) | 0.2336  (0.1701, 0.2971) | 0.2051  (0.1419, 0.2682) |
| Dual elligibility, Yes vs. No | 0.0873  (0.0452, 0.1295) | 0.0936  (0.0517, 0.1355) | 0.1586  (0.1218, 0.1954) | 0.1672  (0.1307, 0.2038) |
| Hospital out patient visits, increase of 1 visit | -0.0031  (-0.0038, -0.0024) | -0.0032  (-0.0039, -0.0026) | -0.003  (-0.0036, -0.0024) | -0.0032  (-0.0038, -0.0026) |
| Home health visits, increase of 1 visit | 0.0055  (0.0049, 0.0060) | 0.0055  (0.0049, 0.0060) | 0.0061  (0.0056, 0.0065) | 0.0061  (0.0056, 0.0066) |
| In patient ER visits, increase of 1 visit | 0.3620  (0.3241, 0.3999) | 0.3691  (0.3315, 0.4068) | 0.3795  (0.3458, 0.4131) | 0.3878  (0.3543, 0.4213) |
| Acute in patient hospital stays, increase of 1 stay | -0.2192  (-0.2585, -0.1799) | -0.2314  (-0.2705, -0.1923) | -0.3347  (-0.3694, -0.3000) | -0.3488  (-0.3833, -0.3142) |
| Skilled nursing facility stays, increase of 1 stay | 0.3801  (0.3515, 0.4087) | 0.3734  (0.3450, 0.4019) | 0.3421  (0.3177, 0.3665) | 0.3362  (0.3119, 0.3605) |
| Hopsice stays, increase of 1 stay | 0.5343  (0.4683, 0.6002) | 0.5374  (0.4717, 0.6031) | 0.5712  (0.5158, 0.6267) | 0.5769  (0.5216, 0.6321) |
| Readmissions, increase of 1 readmission | -0.1423  (-0.188, -0.0966) | -0.1371  (-0.1826, -0.0917) | -0.0925  (-0.1312, -0.0538) | -0.0878  (-0.1264, -0.0493) |
| Current Cognitive functioning impaired, Yes vs. No | 1.2285  (1.1735, 1.2834) | 1.2516  (1.1975, 1.3057) | - | - |
| Confused, Yes vs. No | 1.1843  (1.1233, 1.2453) | 1.1993  (1.1391, 1.2595) | 2.097  (2.0522, 2.1419) | 2.1305  (2.086, 2.175) |
| Anxious, Yes vs. No | -0.2056  (-0.2465, -0.1648) | -0.2052  (-0.2456, -0.1648) | -0.2817  (-0.3183, -0.2452) | -0.2803  (-0.3165, -0.244) |
| Functional dependence, Yes vs. No | 0.6953  (0.6540, 0.7366) | 0.7058  (0.6646, 0.7470) | - | - |
| Anxiety, Yes vs. No | -3.0088  (-3.2167, -2.8009) | -2.944  (-3.1473, -2.7406) | -3.4231  (-3.6148, -3.2315) | -3.3659  (-3.5554, -3.1764) |
| Wandering, Yes vs. No | 1.6182  (1.2648, 1.9716) | 1.6288  (1.2752, 1.9825) | 1.3664  (1.0836, 1.6492) | 1.3695  (1.0892, 1.6498) |
| Cognitive functional awareness impaired, Yes vs. No | -4.9719  (-5.1178, -4.8259) | -4.8954  (-5.0392, -4.7516) | - | - |
| Amnesia, Yes vs. No | 3.2953  (2.9480, 3.6427) | 3.289  (2.9442, 3.6337) | 2.9516  (2.6603, 3.2428) | 2.9379  (2.6474, 3.2284) |
| Executive dysfunction, Yes vs. No | 1.6844  (0.1412, 3.2276) | 1.6560  (0.1195, 3.1925) | 1.6878  (0.3317, 3.0439) | 1.6633  (0.3189, 3.0077) |
| Restlessness and agitation, Yes vs. No | 0.9097  (0.6652, 1.1542) | 0.9344  (0.6911, 1.1776) | 0.5991  (0.4049, 0.7934) | 0.6248  (0.4318, 0.8178) |
| Aphasia, Yes vs. No | 0.3067  (0.1678, 0.4457) | 0.3089  (0.1706, 0.4471) | 0.3876  (0.2649, 0.5103) | 0.3844  (0.2623, 0.5065) |
| Apraxia, Yes vs. No | 1.2267  (0.4867, 1.9667) | 1.2270  (0.4956, 1.9583) | 0.9247  (0.3645, 1.4849) | 0.9129  (0.3564, 1.4695) |
| Depression, Yes vs. No | 0.3324  (0.2937, 0.3712) | 0.3673  (0.3292, 0.4053) | 0.0269  (-0.0065, 0.0602) | 0.0580  (0.0253, 0.0907) |
| Consistent depression medications, Yes vs. No | 0.2352  (0.1462, 0.3242) | - | 0.1688  (0.0945, 0.2431) | - |
| Any dementia medications, Yes vs. No | 1.4464  (1.1597, 1.7330) | - | 1.2687  (1.0346, 1.5028) | - |
| Consistent dementia medications, Yes vs. No | 0.4115  (0.0923, 0.7306) | - | 0.4507  (0.1898, 0.7116) | - |

Intercept terms are included for completeness but are not interpreted. Coefficients are presented to illustrate relative effect size and direction
