# Supplementary material for: Methodological advances in claims-based dementia algorithms: integrating medication and clinical data for medicare populations
Source: BMC Med Res Methodol. 2026 Jun 25;26:207. doi: 10.1186/s12874-026-02929-7 (PMC13573462; doi:10.1186/s12874-026-02929-7)
Supplement: Supplementary file 4 [file 12874_2026_2929_MOESM4_ESM.docx]

| **SUPPLEMENTAL TABLE 3** |  |  |
| --- | --- | --- |
|  | **CCW binary classification algorithm** | **ADRD ternary classification algorithm** |
| **CCW full model** |  |  |
| AUC (95% CI) | 0.8473 (0.8442, 0.8473) | 0.8628 (0.8599, 0.8628) |
| Optimal Threshhold for Performance | 0.600 | 0.625 |
| Sensitivity (95% CI) | 0.7361 (0.7312, 0.7409) | 0.7605 (0.7554, 0.7655) |
| Specificity (95% CI) | 0.8011 (0.7965, 0.8057) | 0.8022 (0.7978, 0.8065) |
| PPV (95% CI) | 0.8018 (0.7972, 0.8064) | 0.7666 (0.7616, 0.7716) |
| NPV (95% CI) | 0.7352 (0.7303, 0.7401) | 0.7967 (0.7923, 0.8011) |
| **CCW model, excluding medication use** |  |  |
| AUC (95% CI) | 0.8439 (0.8408, 0.8439) | 0.8599 (0.857, 0.8599) |
| Optimal Threshhold for Performance | 0.610 | 0.630 |
| Sensitivity (95% CI) | 0.7262 (0.7212, 0.7311) | 0.7545 (0.7494, 0.7595) |
| Specificity (95% CI) | 0.8028 (0.7982, 0.8074) | 0.8019 (0.7975, 0.8062) |
| PPV (95% CI) | 0.8011 (0.7964, 0.8057) | 0.7649 (0.7599, 0.77) |
| NPV (95% CI) | 0.7284 (0.7235, 0.7333) | 0.7926 (0.7882, 0.797) |
| **CCW model, excluding OASIS** |  |  |
| AUC (95% CI) | 0.8301 (0.8268, 0.8301) | 0.8286 (0.8254, 0.8286) |
| Optimal Threshhold for Performance | 0.620 | 0.655 |
| Sensitivity (95% CI) | 0.6899 (0.6848, 0.6951) | 0.6763 (0.6708, 0.6818) |
| Specificity (95% CI) | 0.8022 (0.7976, 0.8068) | 0.8028 (0.7985, 0.8071) |
| PPV (95% CI) | 0.7923 (0.7874, 0.7971) | 0.7456 (0.7402, 0.7509) |
| NPV (95% CI) | 0.703 (0.698, 0.7079) | 0.7438 (0.7392, 0.7483) |
| **CCW model, excluding medication and OASIS** |  |  |
| AUC (95% CI) | 0.8252 (0.8219, 0.8252) | 0.8231 (0.8198, 0.8231) |
| Optimal Threshhold for Performance | 0.625 | 0.660 |
| Sensitivity (95% CI) | 0.6824 (0.6772, 0.6875) | 0.6659 (0.6604, 0.6715) |
| Specificity (95% CI) | 0.8005 (0.7958, 0.8051) | 0.8012 (0.7968, 0.8055) |
| PPV (95% CI) | 0.789 (0.7841, 0.7938) | 0.7411 (0.7356, 0.7465) |
| NPV (95% CI) | 0.6974 (0.6925, 0.7024) | 0.7373 (0.7327, 0.7419) |
| **Novel ADRD full model** |  |  |
| AUC (95% CI) | 0.8132 (0.8097, 0.8132) | 0.906 (0.9037, 0.906) |
| Optimal Threshhold for Performance | 0.555 | 0.480 |
| Sensitivity (95% CI) | 0.7184 (0.7135, 0.7234) | 0.8709 (0.8669, 0.8748) |
| Specificity (95% CI) | 0.8004 (0.7958, 0.8051) | 0.8002 (0.7959, 0.8046) |
| PPV (95% CI) | 0.7974 (0.7927, 0.8021) | 0.7883 (0.7838, 0.7929) |
| NPV (95% CI) | 0.7223 (0.7173, 0.7272) | 0.8788 (0.8751, 0.8826) |
| **Novel ADRD model, excluding medication use** |  |  |
| AUC (95% CI) | 0.8101 (0.8066, 0.8101) | 0.903 (0.9006, 0.903) |
| Optimal Threshhold for Performance | 0.565 | 0.495 |
| Sensitivity (95% CI) | 0.7132 (0.7082, 0.7182) | 0.8638 (0.8597, 0.8678) |
| Specificity (95% CI) | 0.8001 (0.7955, 0.8047) | 0.8023 (0.7979, 0.8066) |
| PPV (95% CI) | 0.796 (0.7913, 0.8007) | 0.7887 (0.7841, 0.7933) |
| NPV (95% CI) | 0.7185 (0.7135, 0.7234) | 0.8733 (0.8695, 0.8771) |
| **Novel ADRD model, excluding OASIS** |  |  |
| AUC (95% CI) | 0.8152 (0.8118, 0.8152) | 0.8527 (0.8497, 0.8527) |
| Optimal Threshhold for Performance | 0.555 | 0.555 |
| Sensitivity (95% CI) | 0.6797 (0.6745, 0.6849) | 0.7435 (0.7384, 0.7487) |
| Specificity (95% CI) | 0.8006 (0.796, 0.8053) | 0.8004 (0.796, 0.8047) |
| PPV (95% CI) | 0.7885 (0.7836, 0.7933) | 0.7609 (0.7558, 0.766) |
| NPV (95% CI) | 0.6957 (0.6907, 0.7007) | 0.785 (0.7806, 0.7895) |
| **Novel ADRD model, excluding medication and OASIS** | |  |
| AUC (95% CI) | 0.8102 (0.8067, 0.8102) | 0.8477 (0.8447, 0.8477) |
| Optimal Threshhold for Performance | 0.565 | 0.565 |
| Sensitivity (95% CI) | 0.6692 (0.664, 0.6744) | 0.7324 (0.7272, 0.7377) |
| Specificity (95% CI) | 0.8003 (0.7956, 0.8049) | 0.8007 (0.7964, 0.8051) |
| PPV (95% CI) | 0.7855 (0.7806, 0.7905) | 0.7585 (0.7533, 0.7636) |
| NPV (95% CI) | 0.6888 (0.6838, 0.6937) | 0.7779 (0.7734, 0.7824) |
